# Trends and Disparities in Alzheimer’s Disease Mortality in the United States: The Impact of COVID-19

**DOI:** 10.3390/neurosci6010016

**Published:** 2025-02-14

**Authors:** Jasleen Chaddha, Eli Blaney, Ali Al-Salahat, Amna Noor, Taylor Billion, Yu-Ting Chen, Abubakar Tauseef, Akshat Sood, Ali Bin Abdul Jabbar

**Affiliations:** 1 School of Medicine, Creighton University, Omaha, NE 68178, USA; jasleenchaddha@creighton.edu (J.C.); eliblaney@creighton.edu (E.B.); taylorbillion@creighton.edu (T.B.); 2 Neurology Department, Creighton University, Omaha, NE 68178, USA; yu-tingchen@creighton.edu; 3 Services Hospital, Lahore 40050, Pakistan; amnanoor101@gmail.com; 4 Department of Medicine, Creighton University, Omaha, NE 68178, USA; abubakartauseef@creighton.edu (A.T.); akshatsood@creighton.edu (A.S.); alibinabduljabbar@creighton.edu (A.B.A.J.)

**Keywords:** Alzheimer’s disease, neurodegenerative diseases, dementia, healthcare disparities, mortality, COVID-19, SARS-CoV-2

## Abstract

**Background:** Alzheimer’s disease (AD) is the fifth leading cause of death for Americans older than 65. Though fluctuations have been noticed over the past two decades, the mortality of Alzheimer’s patients increased considerably during the COVID-19 pandemic. This study aims to explore the temporal trends in AD-associated mortality (ADAM) and disparities in these trends, and we aim to discern changes to these trends during the COVID-19 pandemic. **Methods:** The CDC WONDER Multiple Cause-of-Death Public Use Records from 1999 to 2022 were used to extract population data on deaths related to AD and stratify them based on age, biological sex, race, ethnicity, place of death, census region, and state. ICD-10 codes G30.0, G30.1, G30.8, and G30.9 were used to identify AD-related mortality. Statistical analysis was performed using the Joinpoint Regression Program version 5.0.2. **Results:** We confirmed an increase in mortality rate in all races, sexes, places of death, age groups above 65, and states/census regions. Interestingly, the age-adjusted mortality rate (AAMR) of AD was consistently higher in females compared to males. Non-Hispanic whites had the highest AD mortality by race and ethnicity. At the intersection of race and biological sex, White females had the highest AAMR with AD. Lastly, we noted an increase in AD mortality at hospice facilities as compared to other places of death. **Conclusions:** Our findings demonstrate that the number of deaths due to AD was exacerbated by the recent pandemic and that White females were disproportionately affected. The disparities relating to ADAM uncovered in this study may assist healthcare administrators and policymakers in their decisions. Additionally, the findings might help initiate larger studies focusing on these disparities to explore novel risk/prognostic factors for AD.

## 1. Introduction

Alzheimer’s disease (AD) is a progressive neurodegenerative disorder characterized by cognitive impairment and memory loss [1,2,3]. AD is the sixth leading cause of death for all Americans and the fifth overall cause of death for Americans older than 65 [4]. The number and proportion of Americans with AD are increasing as the population above 65 years is continuing to grow [4]. As of 2023, the prevalence of AD in the United States is 6.7 million Americans—and, of these Americans, 73% are 75 years or older [4]. By 2050, the number of AD patients in the United States is projected to double, to about 13.8 million [3]. The incidence of AD in individuals older than 65 in 2023 was approximately 910,000 [4]. The Alzheimer’s Association Annual Report from 2023 suggests that there will be at least a 6.7% increase in the number of people with AD in each state, with the West and the Southeast having the largest percent increases in cases [4,5]. Additionally, two-thirds of Americans with AD are women, which may be attributed to their longer average lifespan [4]. Data from the Chicago Health and Aging Project (CHAP) suggests that non-Hispanic Black and Hispanic older adults are more likely to develop AD over their lifetime when compared to White older adults [4,5]. The disproportionate impact of AD on these populations highlights the notion that AD is a relevant public health issue with a burden on quality of life.

Even though there have been fluctuations in the prevalence and incidence of AD patients over the past few decades, some of the most extensive changes in these statistics occurred over the SARS-CoV-2 pandemic. Reported deaths from AD increased by more than 145% between 2000 and 2020 [6]. This increased number of AD deaths was likely exacerbated by the COVID-19 pandemic in 2020. The purpose of this study is to analyze the temporal trends and disparities in the mortality of AD, especially during the SARS-CoV-2 pandemic. Specifically, we aim to explore the disparities of race, ethnicity, region, biological sex, age group, and place of death in the mortality of AD. Additionally, we investigated the impact of the COVID-19 pandemic on the mortality trend of AD considering the increased trajectory of deaths noted recently.

## 2. Materials and Methods

Deaths in the United States related to AD were collected using the Centers for Disease Control and Prevention Wide-ranging Online Data for Epidemiologic Research (CDC WONDER) [7]. Institutional Review Board (IRB) approval was not needed as the CDC WONDER database only contains anonymized, publicly available data. The International Classification of Diseases, 10th Revision, Clinical Modification codes G30.0, G30.1, G30.8, and G30.9 were used to identify Alzheimer’s disease-related mortality (ADAM) between 1999 and 2022. Since AD is primarily a diagnosis that affects the elderly population, people under age 65 years old were excluded.

The CDC WONDER Multiple Cause-of-Death Public Use Records from 1999 to 2020 and 2018 to 2022 were used to extract population data on deaths related to AD and stratify based on age, biological sex, race, ethnicity, place of death, census region, and state. Biological sex was defined as female and male. Racial and ethnic groups as identified on death certificates were defined as Hispanic or Latino, non-Hispanic (NH) White, NH Black or African American, and NH American Indian or Alaskan Native. Five-year age groups were defined as 65–69, 70–74, 75–79, 80–84, and 85+ years old. Census regions were defined as Northeast, Midwest, South, and West, consistent with the definitions of the US Census Bureau [8]. Places of death were defined as medical facilities (including inpatient, outpatient, or emergency room, dead on arrival, or other), decedent’s home, hospice, and nursing home or long-term care facility.

Crude mortality rates of AD-related deaths were calculated by dividing the total number of deaths by the United States population. Age-adjusted mortality rate (AAMR) for each stratified group was calculated using the standard population of the United States in 2000. We also extracted total all-cause AAMR in the US over the study period to compare with AD-related AAMR.

Statistical analysis was performed using the Joinpoint Regression Program version 5.0.2 [9]. Significant changes in linear trends of AAMR were identified, and the Joinpoint Monte Carlo permutation test was used to calculate annual percentage change (APC) with a 95% confidence interval (CI) [10]. Significant increases or decreases were determined if the change in mortality over each time interval was significantly different from zero using a 2-tailed *t*-test with α = 0.05.

## 3. Results

### 3.1. Overall

A total of 2,798,698 deaths occurred as a result of AD in the United States over the period of 1999–2022 (Supplemental Table S1). The overall age-adjusted mortality rate (AAMR) in 1999 was 228.0 per 100,000 (95% CI 226.4 to 229.6), increasing to 281.8 per 100,000 (95% CI 280.4 to 283.2) in 2022. The lowest AAMR was in 1999 as above, and the highest AAMR was 329.6 per 100,000 (95% CI 328.0 to 331.1) in 2020. The average annual percentage change (AAPC) during 1999–2022 was 0.88* (95% CI 0.47 to 1.36). Joinpoint analysis calculated four significant periods of change in AAMR (Figure S1): 1999–2005 had an annual percentage change (APC) of 3.56* (95% CI 1.67 to 7.03), decreasing in 2005–2012 to −2.50* (95% CI −6.92 to −0.91), increasing in 2012–2020 to 3.55* (95% CI 2.56 to 7.35), and decreasing in 2020–2022 to −5.33 (95% CI −9.99 to 0.15).

### 3.2. Disparities in AD-Related Mortality by Sex

**Figure 1:**
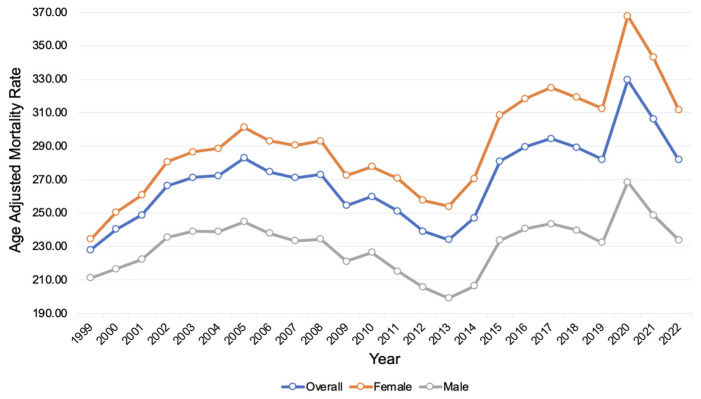
Alzheimer’s disease age-adjusted mortality rates, from 1999 to 2022, stratified by sex. Blue: overall, orange: female, gray: male.

There were 1,919,737 female deaths (68.6%) and 878,961 male deaths (31.4%) related to AD from 1999 to 2022. In females, the AAMR increased from 234.4 (95% CI 232.4 to 236.4) in 1999 to 311.7 (95% CI 309.8 to 313.6) in 2022 (Supplemental Table S2). In males, the AAMR increased from 211.1 (95% CI 208.4 to 213.8) in 1999 to 234.0 (95% CI 231.9 to 236.1) in 2022. The AAPC in females was 1.72* (95% CI 1.26 to 2.42), compared to 0.41* (95% 0.01 to 0.90) in males (Figure 1).

The APC in females during the period of 1999–2004 was 4.76* (95% CI 1.65 to 13.76), decreasing in 2004–2012 to −1.34 (95% CI −6.79 to 0.34), and increasing in 2012–2022 to 2.71* (95% CI 1.58 to 5.40) (Figure S2). In males, the APC during 1999–2005 was 2.40* (95% CI 0.62 to 6.46), decreasing in 2005–2013 to −2.16* (95% CI −6.37 to −0.96), increasing in 2013–2020 to 3.43* (95% CI 2.31 to 7.42), and decreasing in 2020–2022 to −5.33* (95% CI −9.97 to 0.31).

### 3.3. Disparities in AD-Related Mortality by Race and Ethnicity

**Figure 2:**
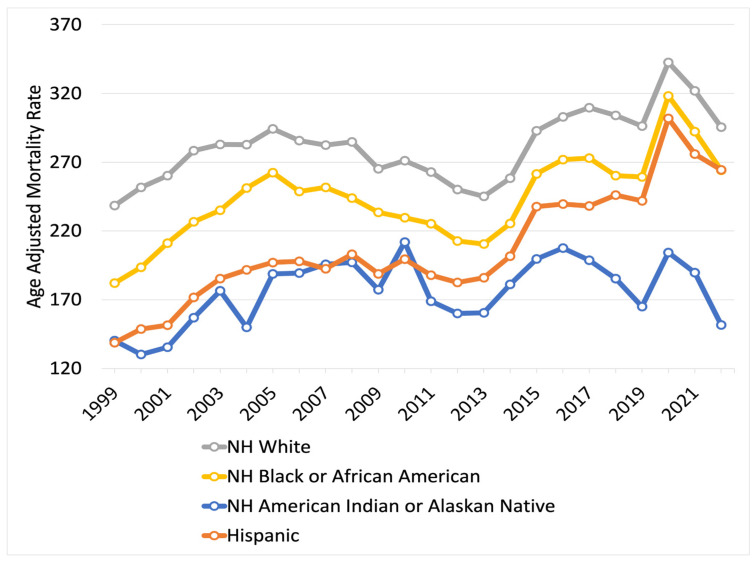
Alzheimer’s disease-related age-adjusted mortality rate, from 1999 to 2022, stratified by race and ethnicity.

People identifying as White had the highest mortality in 1999–2022 related to AD with a total 6759 deaths per 100,000 (31%), followed by 5843 deaths per 100,000 (27%) in Black or African American populations, then 4970 deaths per 100,000 (23%) in Hispanic or Latino populations, and the lowest recorded mortality was 4221 deaths per 100,000 (19%) in those identifying as American Indian or Alaskan Native (Supplemental Table S3). The AAMR of White populations had the greatest rise from 238.49 per 100,000 (95% CI 236.73 to 240.25) in 1999 to 295.40 per 100,000 (95% CI 293.72 to 297.08) in 2022 (Figure 2). The AAMR of Black or African American populations rose from 182.08 (95% CI 176.85 to 187.31) in 1999 to 264.11 (95% CI 259.24 to 268.98) in 2022. The AAMR of Hispanic or Latino populations rose from 138.81 (95% CI 132.30 to 145.32) in 1999 to 264.49 (95% CI 259.68 to 269.30) in 2022. The AAMR of American Indian or Alaskan Native populations rose from 140.23 (95% CI 116.98 to 163.48) in 1999 to 151.59 (95% CI 137.13 to 166.04) in 2022.

The APC in White populations in 1999–2005 was 3.39* (95% CI 1.64 to 6.53), declining in 2005–2012 to −2.36* (95% CI −6.58 to −0.83), rising in 2012–2020 to 3.56* (95% CI 2.58 to 7.27), and falling in 2020–2022 to −5.32 (95% CI −10.06 to 0.29) (Figure S3). In Black or African American populations, the APC in 1999–2005 was 6.13* (95% CI 3.39 to 10.79), falling in 2005–2012 to −2.92* (95% CI −8.25 to −0.72), rising in 2012–2020 to 4.33* (95% CI 3.02 to 9.39), and falling in 2020–2022 to −5.75 (95% CI −11.18 to 1.00). In Hispanic or Latino populations, the AAPC for the total period of 1999–2022 was 2.64* (95% CI 2.09 to 3.40), with no change in trend. In American Indian or Alaskan Native populations, the APC in 1999–2006 was 5.58* (95% CI 1.16 to 28.42), falling in 2006–2022 to −0.32 (95% CI −6.55 to 0.77).

### 3.4. Disparities in AD-Related Mortality by Race and Sex

White females consistently had the highest AAMR among groups stratified by race and biological sex, increasing from 245.24 deaths per 100,000 (95% 243.05 to 247.44) in 1999 to 328.68 deaths per 100,000 (95% CI 326.39 to 330.97) in 2022 (Figure S4, Supplemental Table S4). The APC for White females from 1999 to 2005 was 3.96* (95% CI 2.17 to 7.10), decreasing in 2005–2012 to −2.19* (95% CI −6.46 to −0.64), increasing in 2012–2020 to 4.03* (95% CI 3.03 to 7.71), and decreasing in 2020–2022 to −5.65 (95% CI −10.53 to 0.13) (Figure S5). American Indian or Alaskan Native males consistently had the lowest AAMR, increasing from 115.53 deaths per 100,000 (95% CI 82.53 to 157.31) in 1999 to 116.45 deaths per 100,000 (95% CI 96.70 to 136.19) in 2022. This group had an APC from 1999 to 2010 of 5.56* (95% CI 3.25 to 10.00), decreasing in 2010–2013 to −15.38* (95% CI −21.98 to −1.95), increasing in 2013–2016 to 15.21* (95% CI 1.62 to 25.30), and decreasing in 2016–2022 to −6.22* (95% CI −12.66 to −3.12).

American Indian or Alaskan Native females had an AAMR of 152.59 deaths per 100,000 (95% CI 122.57 to 182.62) in 1999, rising to 177.01 deaths per 100,000 (95% CI 156.53 to 197.50) in 2022. The trend for this group stayed consistent from 1999 to 2022, with an AAPC of 1.04* (95% CI 0.25 to 2.08). Similarly, the AAMR of Hispanic or Latino females rose from 142.18 deaths per 100,000 (95% CI 133.95 to 150.41) in 1999 to 292.22 deaths per 100,000 (95% CI 285.81 to 298.63) in 2022, staying consistent with an AAPC of 2.98 (95% CI 2.37 to 3.84) throughout 1999–2022.

The remaining groups moved in concert with one another from 1999 to 2022, with Black or African American females rising from 183.60 deaths per 100,000 (95% CI 177.23 to 189.98) to 277.33 deaths per 100,000 (95% CI 271.19 to 283.47), Black or African American males rising from 175.68 deaths per 100,000 (95% CI 166.54 to 184.82) to 235.88 deaths per 100,000 (95% CI 227.86 to 243.89), Hispanic males rising from 130.34 deaths per 100,000 (95% CI 119.79 to 140.89) to 217.10 deaths per 100,000 (95% CI 209.98 to 224.22), and White males rising from 220.76 deaths per 100,000 (95% CI 217.82 to 223.70) to 243.86 deaths per 100,000 (95% CI 241.42 to 246.30).

### 3.5. Disparities of AD-Related Mortality by Place of Death

Data regarding the place of death related to AD were included for 2,798,690 deaths (~100% of the total) from 1999 to 2022. A total of 207,602 deaths (7.42%) were attributed to other places of death, and 6219 deaths (0.22%) were attributed to unknown places of death. Of known places of death, 313,147 deaths (11.19%) happened within medical facilities (9.58% inpatient and 1.61% outpatient or emergency room). From 1999 to 2022, inpatient hospital deaths decreased from 13,485 to 9740 (Figure S6). In addition, 5389 deaths (0.19%) were considered dead on arrival at a medical facility, while 717 deaths (0.03%) had an unknown status at a medical facility. A total of 2,265,616 deaths (80.95%) occurred outside of medical facilities, including 1,517,729 deaths (54.23%) at nursing homes or long-term care facilities, 633,473 deaths (22.63%) at the decedent’s home, and 114,414 deaths (4.09%) at hospice facilities. From 1999 to 2022, hospice deaths increased from 9738 to 50,097, with a steeper increase from 40,461 deaths in 2019 to 51,912 deaths in 2020 (Supplemental Table S5).

### 3.6. Disparities in AD-Related Mortality by Age Group

Data were reported for elderly populations between the ages of 65 and 84 since not enough data for the 85+ population group were available. The five-year age group between 80 and 84 years old had the highest overall crude mortality rate (Figure S7), increasing from 368.31 deaths per 100,000 (95% CI 362.93 to 373.69) in 1999 to 422.99 deaths per 100,000 (95% CI 418.05 to 427.93) in 2022 (Supplemental Table S6). The AAPC of this age group was 0.75 (95% CI 0.74 to 0.76). The 75–79 five-year age group experienced a slight increase from 140.99 deaths per 100,000 (95% CI 138.27 to 143.70) in 1999 to 157.22 deaths per 100,000 (95% CI 154.86 to 159.58) in 2022, with an AAPC of 0.66 (95% CI 0.65 to 0.68). The 70–74 five-year age group similarly experienced a slight increase from 47.05 deaths per 100,000 (95% CI 45.63 to 48.48) in 1999 to 53.36 deaths per 100,000 (95% CI 52.20 to 54.52) in 2022, with an AAPC of 0.77 (95% CI of 0.74 to 0.80). The 65–69 five-year age group had the lowest overall crude mortality rate with a slight increase from 14.46 deaths per 100,000 (95% CI 13.70 to 15.23) in 1999 to 17.14 deaths per 100,000 (95% CI 16.55 to 17.74) in 2022, with an AAPC of 0.99 (95% CI 0.94 to 1.04).

### 3.7. Regional Disparities in AD-Related Mortality by State

**Figure 3:**
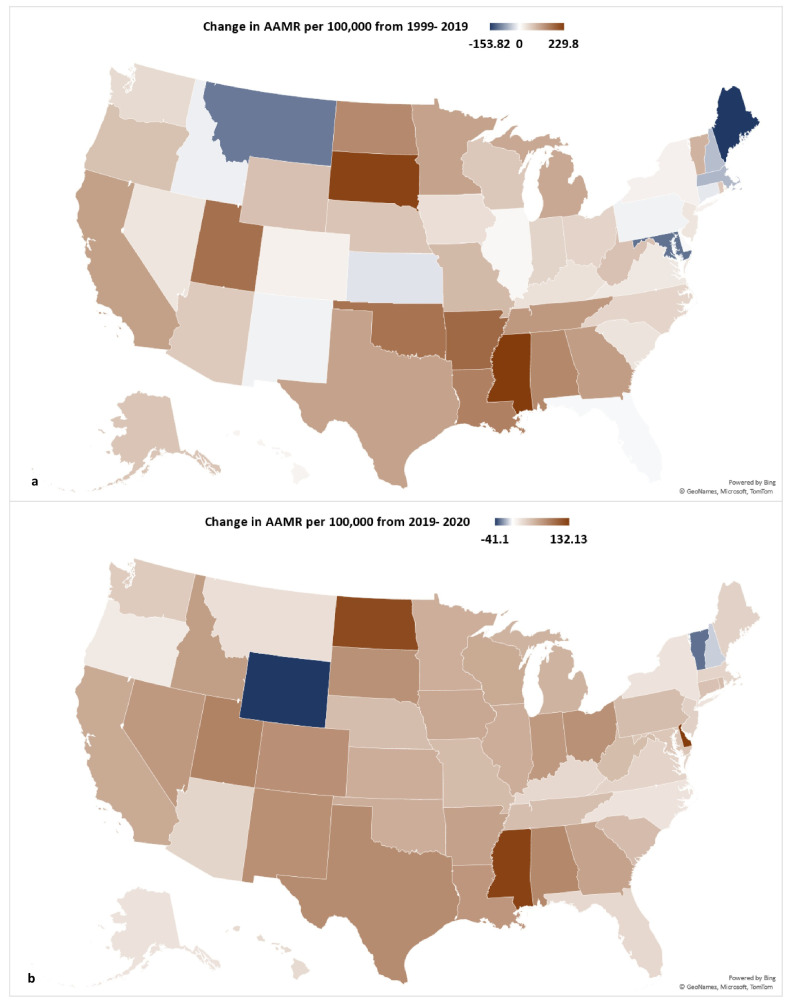
Change in Alzheimer’s disease age-adjusted mortality rate by state from (**a**) 1999 to 2019 (**b**) 2019 to 2020.

The states with the greatest increase in AAMR from 2019 to 2020 were Delaware, Mississippi, and North Dakota (Figure 3). The population in Delaware had an AAMR of 243.10 in 2019 and 375.23 in 2022, demonstrating an increase in AAMR of 132.13 (Supplemental Table S7). Populations in Mississippi and North Dakota similarly experienced an increase in AAMR of 127.62 and 122.32, respectively, over the same time period. Conversely, states that had the greatest decrease in AAMR from 2019 to 2020 include Wyoming, Vermont, and New Hampshire. The population in Wyoming had an AAMR of 300.61 in 2019 and 374.27 in 2020, demonstrating a −41.1 change in AAMR. Over this same period, populations in Vermont and New Hampshire experienced a −29.41 and −9.8 change in AAMR, respectively.

### 3.8. Census-Region Disparities in AD-Related Mortality

An overall increase in AAMR was observed from 1999 to 2022 in the Midwest, South, and West (Figure S8). When comparing these regions, the West consistently had the highest AAMR from 259.08 in 1999 to 344.29 in 2022, with the APC and AAPC consistent at 1.05* (95% CI 0.63 to 1.54) (Supplemental Table S8). An increase in AAMR was observed in the South, from 231.43 in 1999 to 289.96 in 2022 with an AAPC 1.15* (95% CI 0.74 to 1.56). Specifically, the Southern population experienced an increase in APC from 1999 to 2005 at 4.20* (95% CI 2.02 to 8.35), then a decline to −3.10* (95% CI −6.18 to −1.95) from 2005 to 2013, then an increase to 10.28* (95% CI 4.95 to 13.17) from 2013 to 2016, and a decrease to −0.42 (95% CI −3.12 to 0.88) from 2016 to 2022. An increase in AAMR was observed in the Midwest as well, from 237.32 in 1999 to 297.37 in 2022, with an AAPC at 0.93* (95% CI 0.54 to 1.37). The Midwestern population experienced an increase in APC from 1999 to 2005 at 3.24* (95% CI 1.58 to 6.54), then a decline to −1.93* (95% CI −5.57 to −0.79) from 2005 to 2013, then an increase to 4.56* (95% CI 3.45 to 8.12) from 2013 to 2020, and then a decrease to −6.45* (95% CI −11.01 to −1.30) from 2020 to 2022. The Northeast population consistently had the lowest AAMR of all the census regions, with an overall slight decrease in AAMR from 184.3 in 1999 to 175.72 in 2022, with an AAPC of −0.28 (95% CI −0.66 to 0.11). The Northeast population specifically experienced an increase in APC from 1999 to 2004 at 2.65* (95% CI 0.78 to 5.80), then a decline to −2.91* (95% CI −4.87 to −2.06) from 2004 to 2013, then an increase to 2.96* (95% CI 1.92 to 6.13) from 2013 to 2020, and then a decline again to −6.45* (95% CI −10.81 to −5.82) from 2020 to 2022.

### 3.9. Comparison Between Trends of AD-Related AAMR and All-Cause AAMR

**Figure 4:**
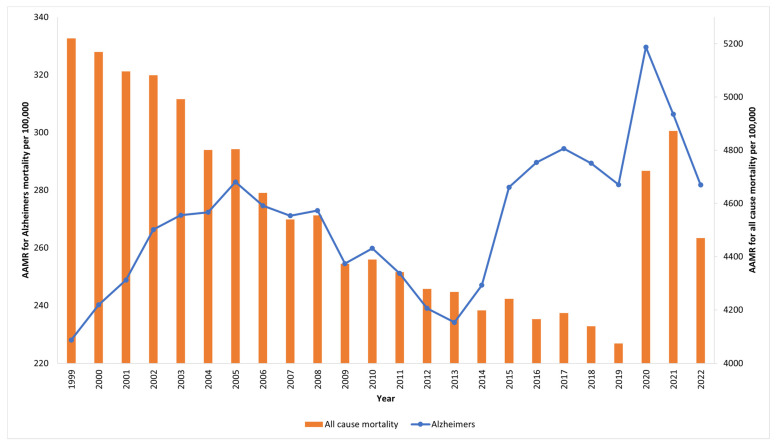
Comparison of Alzheimer’s AAMR to all-cause AAMR for US population ≥65 from 1999 to 2022.

We compared trends of all-cause AAMR in the US over the study period with trends of the total AD-related AAMR. Figure 4 demonstrates the comparison between these trends.

## 4. Discussion

### 4.1. Overall Findings

Our study revealed several notable findings. Overall, ADAM increased from 2019 to 2022, and a particular increase in mortality occurred at the onset of the COVID-19 pandemic from 2019 to 2020. ADAM in females grew at a rate larger than males, with AAPC of mortality in females over four times that of males in the study population. Mortality increased among all races, with the largest increase among White populations and a modest increase among American Indian or Alaskan Native populations. Deaths due to AD were most often in nursing homes or long-term care facilities, though deaths in decedents’ homes have been increasing throughout the study period. Crude mortality was highest in the oldest age group of the study, and each age group had a relatively consistent crude mortality rate throughout the study period. Between 2019 and 2020, the states with the highest individual increase in AAMR were Delaware, Mississippi, and North Dakota, while the states with the largest decrease in AAMR were Wyoming, Vermont, and New Hampshire. The West census region had the greatest AAMR and females from that region had the greatest AAMR when stratified by biological sex.

An increase in AAMR from 1999 to 2022 is observed in all races, sexes, places of death, age groups above 65, and states/census regions. This increase reflects a similar trend to the AAMR analyzed by Zhao et al. and clustered analysis by Mobaderi et al. [11,12]. The AAMR of AD increased by 23% throughout the study period, and joinpoint analysis confirmed an increase during 2012–2020, followed by a decline during 2020–2022. The peak increase of 45% from the start of the study period occurred in 2020. This dramatic excess mortality coincided with the COVID-19 pandemic during a time of increased burden on healthcare systems, even in areas with low COVID-19 prevalence [13,14]. Mattiuzzi & Lippi suggest that this connection may be explained by the increased vulnerability of frail people to infectious diseases, particularly when unvaccinated [15]. Furthermore, scarcities in COVID-19 test availability introduce uncertainty in AD-related mortality due to inadequate testing or inaccurate medical coding. Our study revealed significant disparities in ADAM. Indeed, studies have shown the increasing prevalence of neurodegenerative changes with age, though the acceleration of incidence of AD declines with age [16,17]. Additionally, studies showed pathophysiological interconnections between COVID-19 and AD [18,19,20]. Furthermore, multiple studies from European countries were consistent with our findings, showing a significant link between increased COVID-19 mortality in people with AD [21,22].

### 4.2. Disparities in AD-Related Mortality by Sex

The difference in ADAM by biological sex suggests intrinsic risk factors and modifiable risk factors may interact in the development of AD. Previous studies have shown that women may be intrinsically more predisposed to AD compared to men due to genetic factors, deviations in brain structure and biomarkers, hormonal effects, psychosocial stress responses, vascular disorders, and microglial activation [23,24,25]. Each of these elements must be considered when reviewing disparities in AD risk attributable to biological sex. Extrinsic factors may include sociocultural detection bias and clinical evaluation [4,26]. Cognition in AD and related dementias vary between the sexes, with female patients having stronger performance on verbal memory and processing speed [27]. Further, subjective changes in cognition and memory may differ between males and females; this difference might affect clinical diagnosis and have a different meaning regarding prognosis [28]. Our findings reflect that this disparity remains inadequately accounted for in clinical settings.

### 4.3. Disparities in AD-Related Mortality by Race and Ethnicity

Disparities in ADAM when stratified by race and ethnicity in our study population suggest similar unaccounted risk factors in AD. Previous studies have been consistent with our findings that non-Hospanic White populations have the highest AAMR due to AD [29,30]. Although a neuropathological explanation does not exist for these racial and ethnic disparities, studies have proposed apolipoprotein E ε4 allele status and cultural differences as potential factors [30,31]. *APOE4* genotype status and AD risk have been associated with East Asian and White populations, which is consistent with our findings [32]. Additionally, comorbidities for AD must be considered; hypertension has been proposed as a major contributor to the disparity between White and Black populations with regard to AD risk [33]. Harmful environmental conditions that may be more prevalent in Black communities have also been shown to account for some of the racial and ethnic disparities observed in this study [34,35]. Further study is warranted to determine the effect of risk factors or protective factors associated with race and ethnicity.

### 4.4. Place of Death

Deaths due to AD at nursing homes and long-term care facilities far surpass those at other facilities. Previous studies have shown that patients with AD were more likely to be discharged to lower-quality skilled nursing facilities, and a greater proportion of deaths due to AD in nonmetropolitan areas were in hospitals and nursing facilities [36,37]. Medical inpatient facilities initially had higher mortality than hospice centers, but deaths at inpatient facilities have decreased over time. This inverse relationship has been previously characterized as hospitals have been increasingly transferring terminally ill patients to hospice care, leading to decreased hospital deaths and increased hospice deaths [38]. Further study is warranted to examine the factors that impact the quality of care at locations where patients are transferred and discharged.

### 4.5. Regional Differences

The sharp increase in ADAM from 2019 to 2020 was not uniform among states. While COVID-19 had a global impact, the variation in public health policies in the U.S. has led to disparities in the local impacts of COVID-19 [39]. Population density and similar epidemiological considerations of each state must be taken into account when reviewing the impact of state policies and how these effects may have mitigated or propagated the role of COVID-19 in ADAM. Throughout the study period of 1999–2022, census regions showed similar trends in ADAM. However, the smallest AAMR was observed in the Northeast region, which was consistent in remaining relatively low. When considering these regional differences, educational attainment and health status together may contribute to the diagnosis and prognosis of AD [40].

### 4.6. Limitations

This study has several limitations. Data reporting in the CDC WONDER database may be inaccurate due to missing deaths or missing diagnoses of AD. Causes of death are recorded from death certificates at the state level, where inappropriate identification could underestimate the role of AD over time. Coding biases can further account for differences among the analyzed subgroups. In addition, this study does not claim a statistical cause for any of the associations identified, but these associations may provide a starting point for determining risk factors for AD in future studies. Finally, given that this study is reliant on ICD codes, cases included were probable clinical AD.

## 5. Conclusions

The purpose of this study was to explore the increase in deaths due to AD and analyze the trends in mortality in the United States from 1999–2022 and during the COVID-19 pandemic. We confirmed an increase in mortality rate in all races, sexes, places of death, age groups above 65, and states/census regions. Interestingly, we also found that the AAMR of AD was consistently higher in females compared to males. Additionally, we found that non-Hispanic whites had the highest AD mortality by race and ethnicity. At the intersection of race and biological sex, White females had the highest AAMR with AD. Lastly, we noted nursing homes and long-term care facilities had the most deaths due to AD when compared to other places of death. Our findings demonstrate not only that the number of deaths due to AD was exacerbated by the recent pandemic, but also that certain subgroups of AD patients were disproportionately affected. The disparities relating to ADAM uncovered in this study may assist healthcare administrators and policymakers in their decisions. Additionally, the findings might help initiate larger studies focusing on these disparities to explore novel risk/prognostic factors for AD.

## Data Availability

The data compiled in this manuscript are publicly available upon request from the CDC WONDER database.

## Supplementary Materials

The following supporting information can be downloaded at: https://www.mdpi.com/article/10.3390/neurosci6010016/s1, Figure S1: overall AAMR for deaths related to Alzheimer’s disease from 1999 to 2022. Figure S2: APC of deaths due to Alzheimer’s disease from 1999 to 2022, stratified by biological sex. Blue, overall; green, female; gray, male. Figure S3: APC of deaths due to Alzheimer’s disease from 1999 to 2022, stratified by race and ethnicity. Figure S4: AAMR of deaths relating to Alzheimer’s disease from 1999 to 2022, stratified by race/ethnicity and biological sex. Figure S5: APC of deaths relating to Alzheimer’s disease from 1999 to 2022, stratified by biological sex and race/ethnicity. Figure S6: deaths related to Alzheimer’s disease from 1999 to 2022, stratified by place of death. Figure S7: crude mortality rate of deaths related to Alzheimer’s disease from 1999 to 2022, stratified by 5-year age groups. Figure S8: AAMR of deaths related to Alzheimer’s disease from 1999 to 2022, stratified by census region. Figure S9: APC of deaths related to Alzheimer’s disease from 1999 to 2022, stratified by census region and biological sex. Table S1: crude number of deaths due to Alzheimer’s disease stratified by biological sex, race, census region, and age group from 1999 to 2022. Table S2: AAMR of deaths due to Alzheimer’s disease stratified by biological sex from 1999 to 2022. Table S3: AAMR of deaths due to Alzheimer’s disease stratified by race from 1999 to 2022. Table S4: AAMR of deaths due to Alzheimer’s disease stratified by race and biological sex from 1999 to 2022. Table S5: crude number of deaths due to Alzheimer’s disease stratified by place of death from 1999 to 2022. Table S6: AAMR of deaths due to Alzheimer’s disease stratified by age group from 1999 to 2022. Table S7: AAMR of deaths due to Alzheimer’s disease stratified by state in 1999, 2019, and 2020. Table S8: AAMR of deaths due to Alzheimer’s disease stratified by census region from 1999 to 2022. Table S9: AAMR of deaths due to Alzheimer’s disease stratified by census region and biological sex from 1999 to 2022.

## Abbreviations

Parkinson’s Disease (PD), CDC Wide-ranging Online Data for Epidemiologic Research (CDC WONDER), Age-adjusted Mortality Rate (AAMR), International Classification of Diseases (ICD), Non-Hispanic (NH), Annual Percent Change (APC), Confidence Interval (CI), Average Annual Percent Change (AAPC).
