# Supplementary material for: Trends and Disparities in Alzheimer’s Disease Mortality in the United States: The Impact of COVID-19
Source: NeuroSci. 2025 Feb 14;6(1):16. doi: 10.3390/neurosci6010016 (PMC11843863; doi:10.3390/neurosci6010016)
Supplement: Supplementary file 1 [file neurosci-06-00016-s001.zip › neurosci-3455291-supplementary.pdf]

NeuroSci

Supplementary

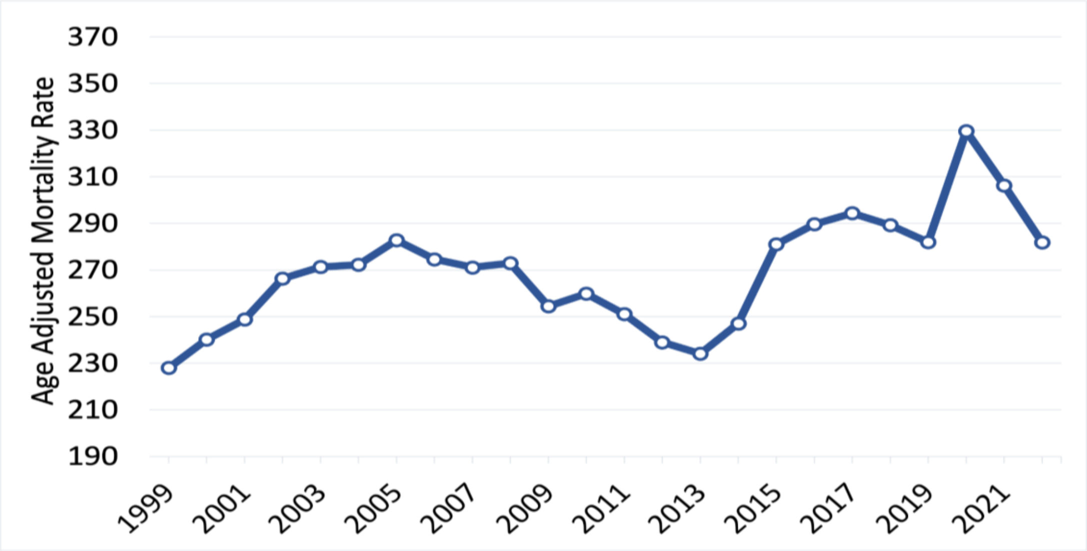

Figure S1. Overall AAMR for deaths related to Alzheimer's disease from 1999–2022.

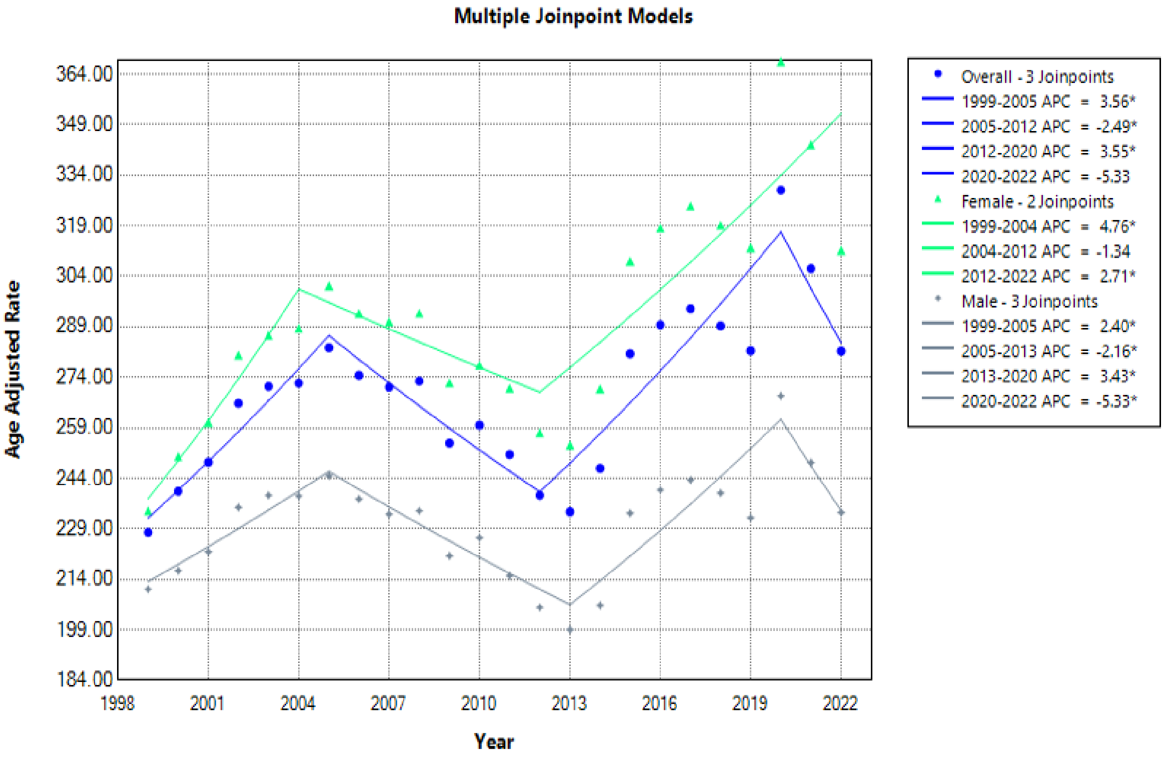

Figure S2. APC of deaths due to Alzheimer's disease from 1999–2022, stratified by biological sex. Blue, overall; green, female; gray, male;.

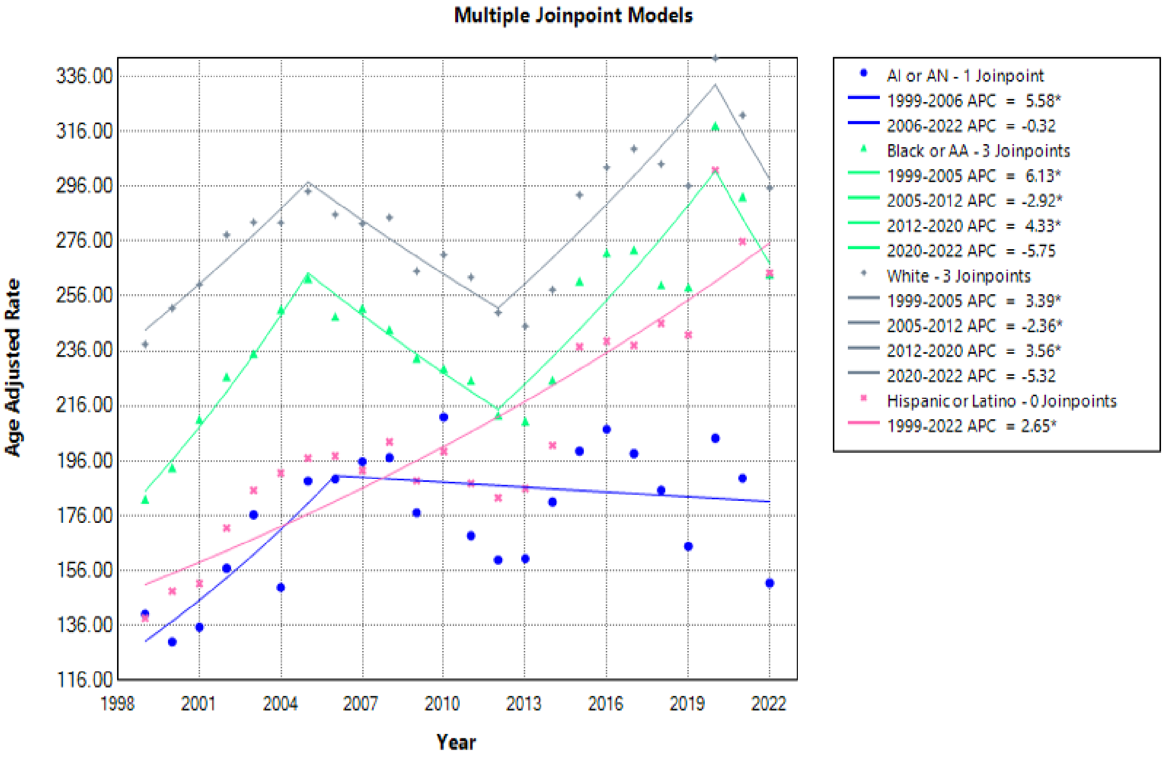

Figure S3. APC of deaths due to Alzheimer's disease from 1999–2022, stratified by race and ethnicity. Blue, non-Hispanic American Indian or Alaskan Native; green, non-Hispanic Black or African American; gray, non-Hispanic White; pink, Hispanic or Latino.

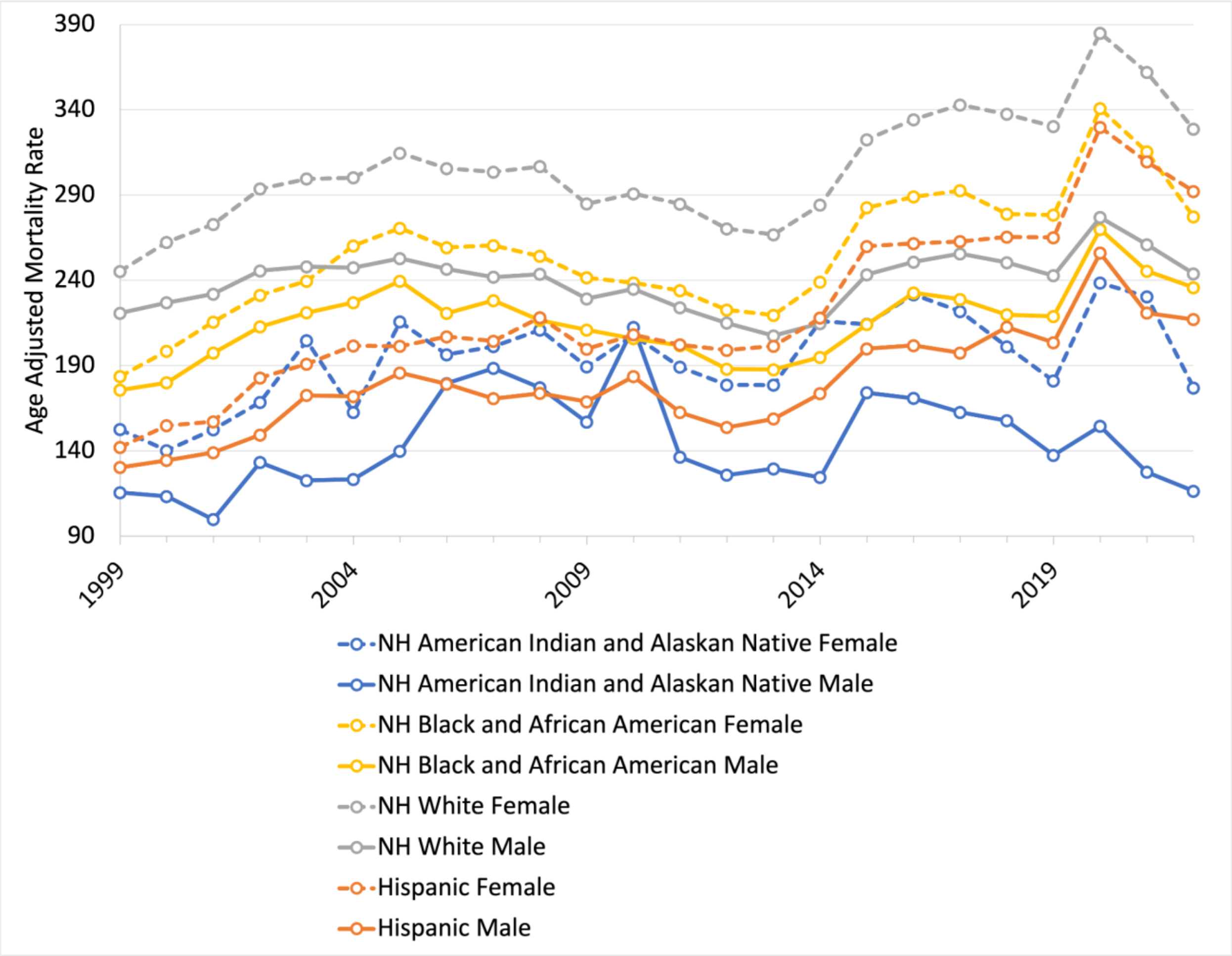

Figure S4. AAMR of deaths relating to Alzheimer's disease from 1999–2022, stratified by race/ethnicity and biological sex. Dashed, female; solid, male. Blue, non-Hispanic White; orange, non-Hispanic Black or African American; gray, non-Hispanic American Indian or Alaskan Native; yellow, Hispanic.

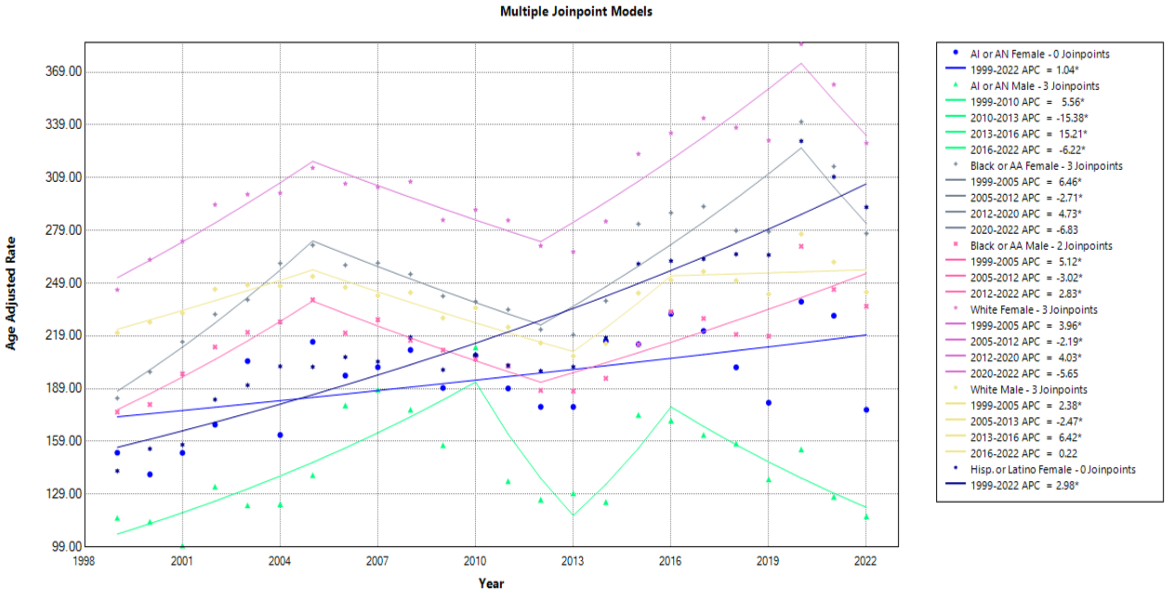

Figure S5. APC of deaths relating to Alzheimer's disease from 1999–2022, stratified by biological sex and race/ethnicity. Blue, non-Hispanic American Indian or Alaskan Native female; green, non-Hispanic American Indian or Alaskan Native male; gray, non-Hispanic Black or African American female; pink, non-Hispanic Black or African American male; magenta, non-Hispanic White female; yellow, non-Hispanic White male; indigo, Hispanic or Latino female.

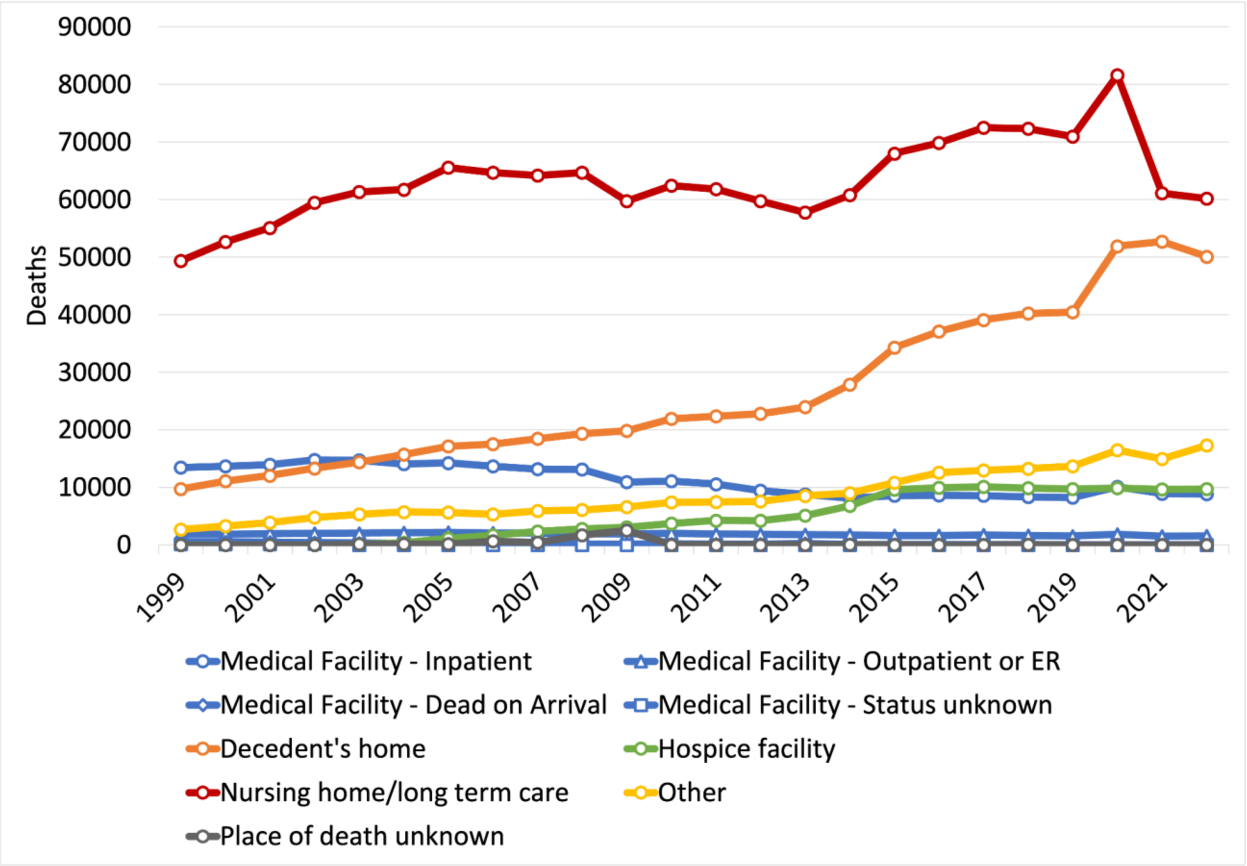

Figure S6. Deaths related to Alzheimer's disease from 1999–2022, stratified by place of death. Blue circle, medical facility - inpatient; blue triangle, medical facility - outpatient or emergency room; blue diamond, medical facility - dead on arrival; blue square, medical facility - status unknown;

orange circle, decedent's home; green circle, hospice facility; red circle, nursing home or long-term care facility; yellow, other place of death; gray, unknown place of death.

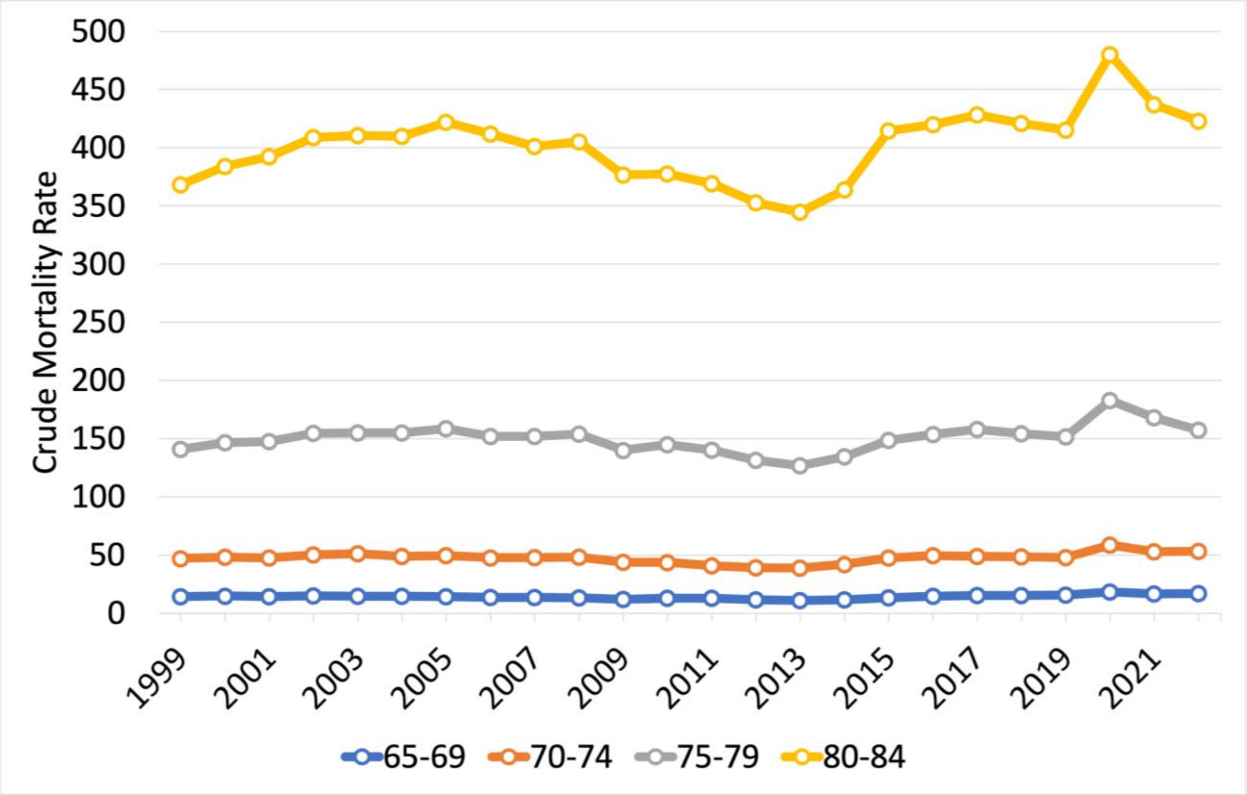

Figure S7. Crude mortality rate of deaths related to Alzheimer's disease from 1999–2022, stratified by 5-year age groups. Blue, 65–69; orange, 70–74; gray, 75–79; yellow, 80–84.

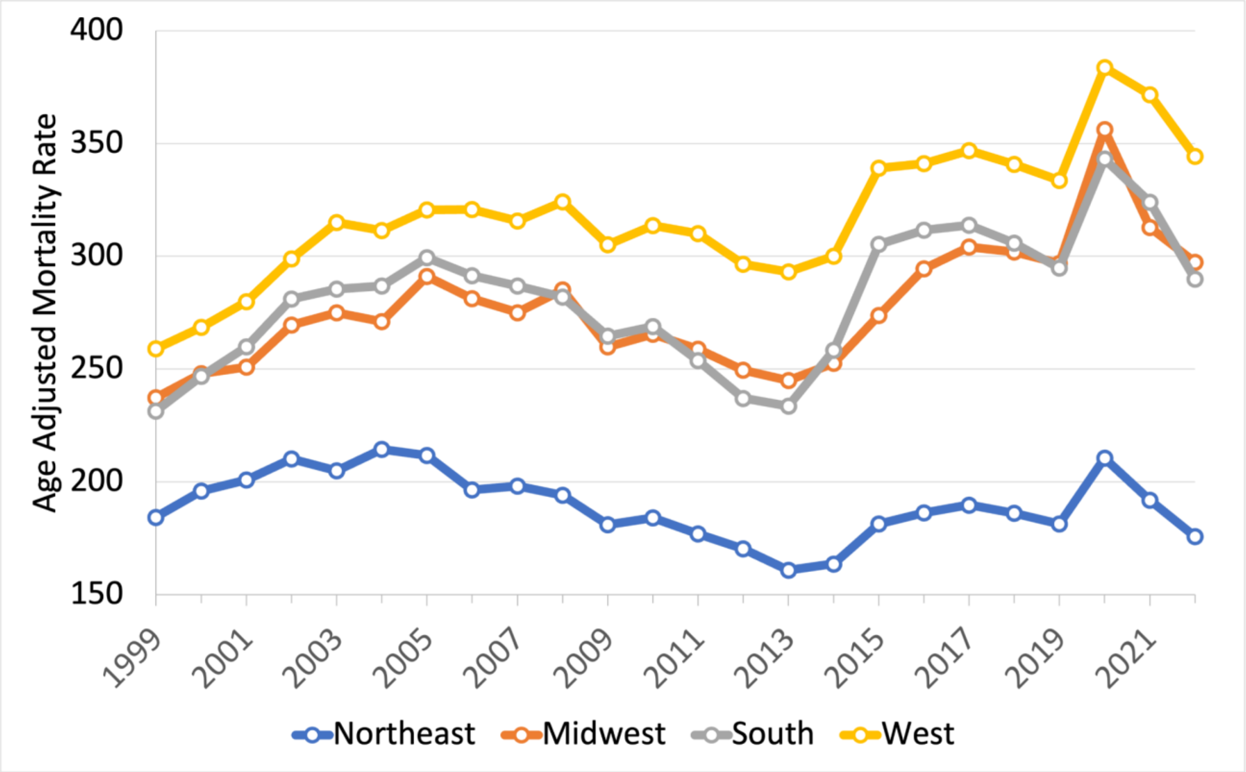

Figure S8. AAMR of deaths related to Alzheimer's disease from 1999–2022, stratified by census region. Blue, Northeast; orange, Midwest; gray, South; yellow, West.

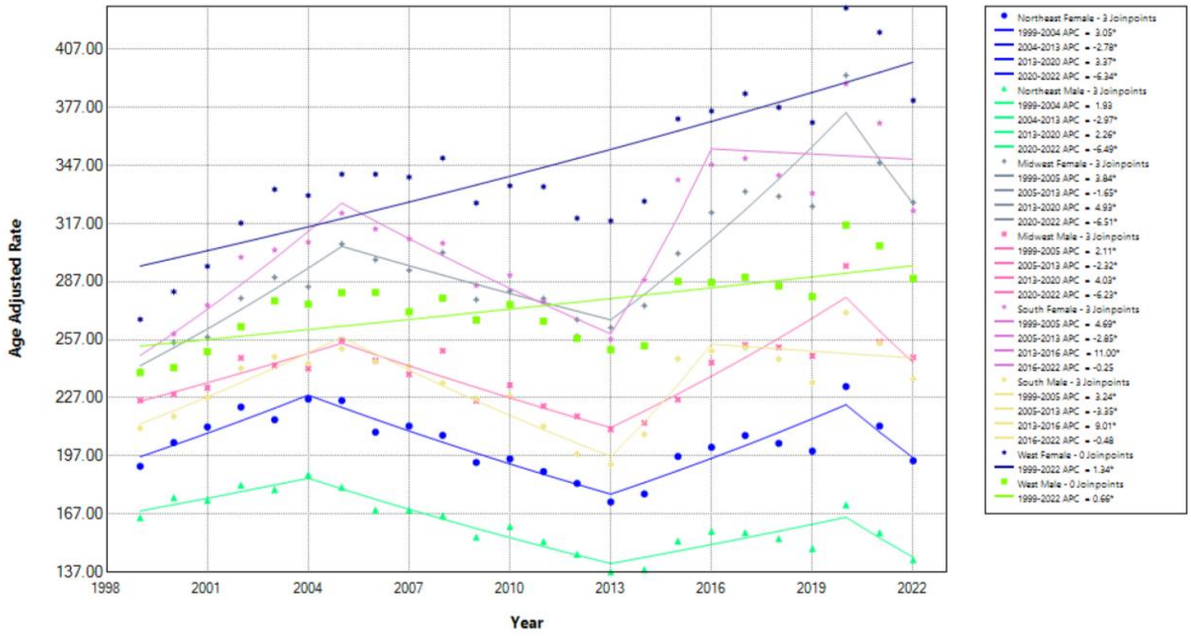

Figure S9. APC of deaths related to Alzheimer's disease from 1999–2022, stratified by census region and biological sex. Blue, Northeast female; green triangles, Northeast male; gray, Midwest female; pink, Midwest male; magenta, South female; yellow, South Male; indigo, West female; green squares, West male.

Table S1. Crude number of deaths due to Alzheimer's disease stratified by biological sex, race, census region, and age group from 1999–2022.

Year	Overall	Female	Male	NH White	NH Black	NH American Indian or Alaskan Native	Hispanic	Northeast	Midwest	South	West	65-69	70-74	75-79	80-84	85-89	90-94	95-99	100+
1999	77885	53174	24711	70647	4676	143	1780	13729	19980	27165	17011	1380	4177	10352	17982	21372	15677	5971	974
2000	83342	57615	25727	75462	5026	149	2004	14844	21083	29382	18033	1412	4279	10873	18983	23011	17162	6504	1118
2001	87740	60780	26960	79159	5497	155	2170	15454	21642	31318	19326	1354	4184	11016	20165	24811	18029	7011	1170
2002	95103	66057	29046	85578	5921	181	2554	16382	23491	34179	21051	1456	4404	11546	21649	26851	19933	7919	1345
2003	98527	68359	30168	88268	6191	205	2927	16247	24320	35217	22743	1433	4437	11632	22155	27620	21329	8504	1417
2004	100245	69514	30731	89191	6681	180	3158	17172	24246	35878	22949	1468	4200	11566	22667	28187	21973	8640	1544
2005	106497	74005	32492	94503	7163	230	3474	17291	26439	38354	24413	1471	4272	11835	23676	30171	23719	9660	1693
2006	105889	73386	32503	93726	6912	244	3688	16345	26066	38296	25182	1431	4128	11316	23257	30458	24128	9531	1640
2007	106964	74140	32824	94421	7175	263	3807	16792	25988	38623	25561	1511	4170	11236	22812	31029	24461	9963	1782
2008	109929	76030	33899	96768	7160	271	4243	16712	27315	38874	27028	1558	4299	11317	23224	32027	25167	10486	1851
2009	104805	72013	32792	91726	7051	256	4191	15869	25298	37389	26249	1459	4008	10217	21569	31121	24323	10178	1930
2010	108888	74553	34335	95114	7128	317	4639	16326	26196	38746	27620	1597	4053	10597	21682	32212	25836	10895	2016
2011	108709	74721	33988	94604	7229	270	4783	16043	26147	38000	28519	1664	3935	10368	21373	31634	26421	11228	2086
2012	105857	72368	33489	91432	7124	273	4963	15694	25572	36543	28048	1629	3912	9844	20393	30242	26497	11128	2212
2013	106291	72777	33514	91212	7293	288	5424	15039	25588	37170	28494	1603	4130	9740	19879	30455	26827	11434	2223
2014	114528	78718	35810	97422	8078	349	6308	15494	26732	42226	30076	1786	4652	10656	20957	31679	29576	12614	2608
2015	133115	91386	41729	111953	9730	407	7947	17366	29350	51376	35023	2119	5472	12064	24048	36147	34798	15324	3143
2016	139893	95824	44069	117356	10405	454	8466	18040	31851	53837	36165	2444	5837	12870	24622	37730	36332	16656	3402

2017	145126	99157	45969	121423	10818	455	8936	18887	33460	55490	37289	2598	6280	13813	25545	38274	36929	17960	3727
2018	145866	99196	46670	121257	10696	458	9696	18676	33720	55740	37730	2628	6506	14294	25797	37744	36977	18168	3752
2019	144734	98232	46502	119593	11002	427	9847	18316	33511	55137	37770	2724	6706	14640	26222	37016	35780	17851	3795
2020	171976	116906	55070	139540	13921	571	12893	21354	40378	65851	44393	3308	8602	18278	31024	43265	41898	21132	4469
2021	148972	100893	48079	120184	12016	478	11444	18363	32784	58019	39806	3085	8083	16661	27537	37161	35262	17552	3631
2022	147817	99933	47884	119061	11451	427	11706	17864	33371	56356	40226	3193	8088	17075	28166	36746	33857	16964	3728
**Total**	**2798698**	**1919737**	**878961**	**2399600**	**196344**	**7451**	**141048**	**404299**	**664528**	**1029166**	**700705**	**46311**	**122814**	**293806**	**555384**	**766963**	**662891**	**293273**	**57256**

Table S2. AAMR of deaths due to Alzheimer's disease stratified by biological sex from 1999–2022.

Year	Overall	Female	Male
1999	228.00	234.40	211.10
2000	240.20	250.50	216.60
2001	248.80	260.60	222.20
2002	266.30	280.60	235.40
2003	271.30	286.50	239.00
2004	272.30	288.60	238.80
2005	282.80	301.20	244.80
2006	274.60	293.00	237.90
2007	271.10	290.50	233.40
2008	272.90	293.10	234.40
2009	254.50	272.40	221.00
2010	259.80	277.60	226.40
2011	251.10	270.80	215.10
2012	239.00	257.60	205.70
2013	234.10	253.90	199.10
2014	247.00	270.60	206.30
2015	281.00	308.50	233.70
2016	289.60	318.40	240.60
2017	294.40	324.90	243.50
2018	289.30	319.20	239.70
2019	281.90	312.50	232.30
2020	329.60	367.80	268.50
2021	306.30	343.10	248.70
2022	281.80	311.70	233.90

Table S3. AAMR of deaths due to Alzheimer's disease stratified by race from 1999–2022.

Year	Overall	NH White	NH Black or African American	NH American Indian or Alaskan Native	Hispanic
1999	228.00	238.49	182.08	140.23	138.81
2000	240.20	251.59	193.53	130.17	148.60
2001	248.80	260.20	211.19	135.48	151.40
2002	266.30	278.34	226.69	156.94	171.60
2003	271.30	282.87	235.08	176.40	185.33
2004	272.30	282.76	251.24	149.93	191.63
2005	282.80	294.16	262.42	188.69	197.05
2006	274.60	285.71	248.66	189.41	197.84
2007	271.10	282.34	251.59	195.74	192.54
2008	272.90	284.67	243.85	197.19	202.99
2009	254.5	265.09	233.41	177.19	188.82
2010	259.80	271.05	229.63	211.96	199.45
2011	251.10	262.88	225.32	168.80	187.81
2012	239.00	250.06	212.68	159.97	182.57
2013	234.10	245.07	210.52	160.44	185.98
2014	247.00	258.26	225.41	181.06	201.67
2015	281.00	292.82	261.51	199.57	237.60
2016	289.60	302.88	271.89	207.51	239.60
2017	294.40	309.60	272.86	198.67	238.10
2018	289.30	304.03	260.14	185.37	246.05
2019	281.90	296.18	259.26	164.92	241.92
2020	329.60	342.59	318.10	204.27	301.80
2021	306.30	321.82	292.25	189.72	275.89
2022	281.80	295.39	264.02	151.59	264.45

Table S4. AAMR of deaths due to Alzheimer's disease stratified by race and biological sex from 1999–2022.

Year	NH American Indian or Alaskan Native Female	NH American Indian or Alaskan Native Male	NH Black or African American Female	NH Black or African American Male	NH White Female	NH White Male	Hispanic Female	Hispanic Male
1999	152.59	115.53	183.60	175.68	245.24	220.76	142.18	130.34
2000	140.26	113.31	198.53	179.99	262.38	226.87	154.81	134.44
2001	152.50	99.77	215.65	197.42	272.85	232.00	157.11	138.99
2002	168.52	133.25	231.29	212.73	293.65	245.59	182.83	149.33
2003	204.67	122.62	239.58	221.03	299.49	248.05	190.97	172.59
2004	162.64	123.29	260.30	226.97	300.22	247.43	201.68	171.97
2005	215.66	139.79	270.62	239.58	314.59	252.86	201.41	185.79
2006	196.46	179.52	259.28	220.67	305.61	246.65	206.94	179.26
2007	201.21	188.45	260.46	228.20	303.60	241.93	204.38	170.68
2008	210.99	177.02	254.12	216.52	306.76	243.67	218.21	173.81
2009	189.41	156.86	241.59	210.95	284.87	229.22	199.75	168.85
2010	207.84	212.54	238.56	205.84	290.81	234.94	208.11	183.60
2011	189.15	136.38	233.97	201.83	284.74	223.97	202.2	162.58
2012	178.67	125.84	222.65	188.00	270.24	214.99	199.08	153.80
2013	178.64	129.51	219.61	187.63	266.86	207.51	201.44	158.79
2014	216.02	124.48	238.95	194.87	284.17	214.74	217.95	173.6
2015	214.29	174.09	282.62	214.17	322.52	243.37	259.97	199.92
2016	231.60	170.87	288.99	232.67	334.31	250.80	261.67	201.83
2017	221.83	162.69	292.60	228.92	342.85	255.62	262.76	197.47

2018	201.15	157.77	278.87	219.84	337.48	250.43	265.43	212.60
2019	181.02	137.50	278.29	218.84	330.21	242.75	265.07	203.56
2020	238.48	154.48	340.76	269.98	385.00	276.95	329.82	256.05
2021	230.48	127.54	315.38	245.45	361.95	261.00	309.48	220.87
2022	177.01	116.45	277.27	235.81	328.65	243.86	292.22	217.06

Table S5. Crude number of deaths due to Alzheimer's disease stratified by place of death from 1999–2022.

Year	Medical Facility - Inpatient	Medical Facility - Outpatient or ER	Medical Facility - Dead on Arrival	Medical Facility - Status unknown	Decedent's home	Hospice facility	Nursing home or long-term care	Other	Place of death unknown
1999	13485	1868	535	165	9738	Missing	49371	2708	15
2000	13688	1889	538	161	11102	Missing	52639	3313	14
2001	13954	1981	513	189	12068	Missing	55099	3907	29
2002	14798	2048	432	202	13330	Missing	59465	4802	26
2003	14726	2067	359	0	14390	172	61349	5311	153
2004	14059	2147	293	0	15721	372	61748	5747	158
2005	14266	2213	275	0	17128	1171	65591	5680	173
2006	13677	2084	245	0	17516	1684	64696	5323	664
2007	13204	2071	259	0	18468	2380	64174	5962	446
2008	13121	1962	172	0	19353	2825	64705	6106	1685
2009	10931	1946	153	0	19847	3066	59757	6591	2514
2010	11101	2040	196	0	21910	3744	62459	7407	31
2011	10585	1923	174	0	22394	4260	61854	7489	30
2012	9437	1884	167	0	22797	4219	59747	7578	28
2013	8776	1832	264	0	23958	5097	57781	8550	33
2014	8140	1751	136	0	27855	6819	60772	9000	55
2015	8561	1649	130	0	34298	9615	68011	10822	29
2016	8615	1665	104	0	37086	9959	69869	12580	15
2017	8574	1746	85	0	39104	10127	72471	12992	27
2018	8358	1667	83	0	40255	9879	72309	13273	42
2019	8233	1567	72	0	40461	9729	70961	13691	19
2020	10097	1871	81	0	51912	9900	81599	16497	19
2021	8932	1534	63	0	52685	9656	61132	14956	14
2022	8839	1585	60	0	50097	9740	60171	17317	0

Table S6. AAMR of deaths due to Alzheimer's disease stratified by age group from 1999–2022.

Year	65-69	70-74	75-79	80-84
1999	14.46	47.05	140.99	368.31
2000	14.81	48.31	146.62	383.85
2001	14.16	47.43	147.76	392.45
2002	15.11	50.33	154.56	408.90
2003	14.58	51.18	155.14	410.38
2004	14.57	48.87	155.01	410.00
2005	14.31	49.66	158.57	421.94
2006	13.56	47.72	151.95	411.77
2007	13.77	47.77	151.83	401.12
2008	13.44	48.24	154.06	405.28
2009	12.07	43.85	140.05	376.57
2010	12.84	43.68	144.81	377.52
2011	12.93	40.96	140.32	369.36
2012	11.65	39.09	131.44	352.63
2013	10.97	38.93	126.86	344.60
2014	11.65	42.01	134.51	363.81
2015	13.19	47.65	148.50	414.67
2016	14.53	49.42	153.80	419.77
2017	15.43	48.88	158.02	428.23
2018	15.38	48.53	154.25	421.02
2019	15.61	47.80	151.67	415.09
2020	18.51	58.61	183.02	479.9
2021	16.77	52.93	168.21	437.00
2022	17.14	53.36	157.21	422.94

Table S7. AAMR of deaths due to Alzheimer's disease stratified by state in 1999, 2019, and 2020.

State	1999	2019	2020
Alabama	241.43	380.91	461.07
Alaska	172.73	241.95	261.18
Arizona	226.57	288.52	316.73
Arkansas	184.54	360.40	423.85
California	239.82	350.49	407.94
Colorado	311.80	328.40	403.21
Connecticut	190.40	173.09	214.84
Delaware	247.10	243.10	375.23
District of Columbia	162.62	132.11	132.00
Florida	174.90	169.81	195.67
Georgia	261.99	376.56	439.05
Hawaii	170.58	184.06	208.83
Idaho	317.59	305.49	371.05
Illinois	215.54	224.92	279.98
Indiana	257.04	307.52	376.56
Iowa	254.65	293.27	351.74
Kansas	229.90	208.70	263.91
Kentucky	269.24	304.27	330.32
Louisiana	218.05	365.92	436.41
Maine	386.65	232.83	263.65
Maryland	249.26	140.45	178.71
Massachusetts	221.02	165.45	194.83
Michigan	221.57	323.12	373.96
Minnesota	233.04	342.41	396.72
Mississippi	207.81	437.61	565.23
Missouri	236.00	317.71	363.39
Montana	321.07	217.93	239.96
Nebraska	225.18	294.22	338.78
Nevada	169.38	199.30	268.20
New Hampshire	317.31	267.05	257.25
New Jersey	173.12	203.16	234.33
New Mexico	224.91	214.89	289.97
New York	125.14	141.96	160.78
North Carolina	288.91	338.49	357.38
North Dakota	228.93	366.95	489.27

Ohio	259.95	310.88	385.31
Oklahoma	221.68	385.92	440.91
Oregon	294.98	367.97	382.24
Pennsylvania	211.09	201.06	245.51
Rhode Island	215.58	280.70	326.29
South Carolina	311.22	343.12	388.52
South Dakota	217.48	437.90	512.14
Tennessee	262.52	382.56	426.10
Texas	254.95	364.15	441.54
Utah	200.37	368.32	451.93
Vermont	282.39	371.74	342.33
Virginia	215.12	243.34	273.00
Washington	373.98	417.57	452.54
West Virginia	234.06	306.21	350.91
Wisconsin	246.62	311.07	368.21
Wyoming	300.61	374.27	333.17

Table S8. AAMR of deaths due to Alzheimer's disease stratified by census region from 1999–2022.

Year	Northeast	Midwest	South	West
1999	184.30	237.32	231.43	259.08
2000	195.95	247.97	246.77	268.53
2001	200.86	250.82	259.90	279.85
2002	210.12	269.53	281.05	298.95
2003	204.85	274.91	285.54	314.92
2004	214.40	271.05	286.78	311.37
2005	211.75	291.10	299.33	320.64
2006	196.41	281.21	291.41	320.70
2007	198.07	275.04	286.89	315.67
2008	194.00	285.14	281.88	324.10
2009	180.94	259.79	264.60	305.19
2010	183.99	265.47	268.88	313.66
2011	176.85	258.90	253.80	310.06
2012	170.28	249.46	237.05	296.47
2013	160.80	244.97	233.56	293.12
2014	163.61	252.58	258.26	300.13
2015	181.35	273.82	305.28	339.02
2016	186.23	294.38	311.55	341.11
2017	189.73	304.20	313.72	346.8
2018	186.02	301.95	305.77	340.74
2019	181.27	296.78	294.84	333.69
2020	210.51	356.14	343.18	383.68
2021	191.84	312.83	324.04	371.55
2022	175.72	297.37	289.96	344.29

Table S9. AAMR of deaths due to Alzheimer's disease stratified by census region and biological sex from 1999–2022.

Year	Northeast Female	Northeast Male	Midwest Female	Midwest Male	South Female	South Male	West Female	West Male
1999	191.56	165.11	240.68	225.57	239.53	211.26	267.50	240.01
2000	203.83	175.50	255.49	228.77	260.00	217.33	281.78	242.47
2001	211.92	173.99	258.33	232.13	274.83	226.94	294.92	250.88
2002	222.19	181.87	278.44	247.57	299.62	242.21	317.30	263.75
2003	215.64	179.58	289.24	243.63	303.36	248.14	334.72	276.99
2004	226.29	187.25	284.36	242.12	307.41	244.67	331.53	275.25
2005	225.55	180.75	306.48	256.89	322.43	252.32	342.55	281.21
2006	209.23	168.98	298.34	246.15	314.25	245.71	342.49	281.40
2007	212.40	168.97	292.88	239.19	309.23	243.28	341.02	271.42
2008	207.59	166.02	302.03	251.27	306.83	234.69	350.80	278.49
2009	193.58	155.04	277.70	225.40	285.06	226.71	327.67	267.11
2010	195.40	160.52	282.21	233.48	290.43	227.94	336.56	275.16
2011	188.85	152.71	278.28	222.71	276.66	212.21	336.10	266.60
2012	182.75	146.18	267.32	217.42	259.01	197.96	319.78	257.74
2013	173.15	137.02	263.21	210.64	257.26	192.64	318.43	251.83
2014	177.29	138.21	274.6	214.00	287.84	208.09	328.57	253.80
2015	196.65	153.06	301.56	226.06	339.62	247.17	371.13	287.02
2016	201.40	158.00	322.69	245.21	347.51	251.16	375.12	286.64
2017	207.47	157.36	333.56	254.34	350.70	252.78	384.14	289.25
2018	203.45	154.24	331.06	253.09	341.94	247.00	377.04	284.65
2019	199.45	149.17	325.85	248.64	332.67	234.98	369.23	279.30
2020	232.79	171.74	393.64	295.15	389.21	271.04	428.46	316.18
2021	212.40	157.20	348.49	255.89	368.90	255.22	415.84	305.63
2022	194.46	143.24	327.92	247.84	323.64	236.74	380.65	288.65
